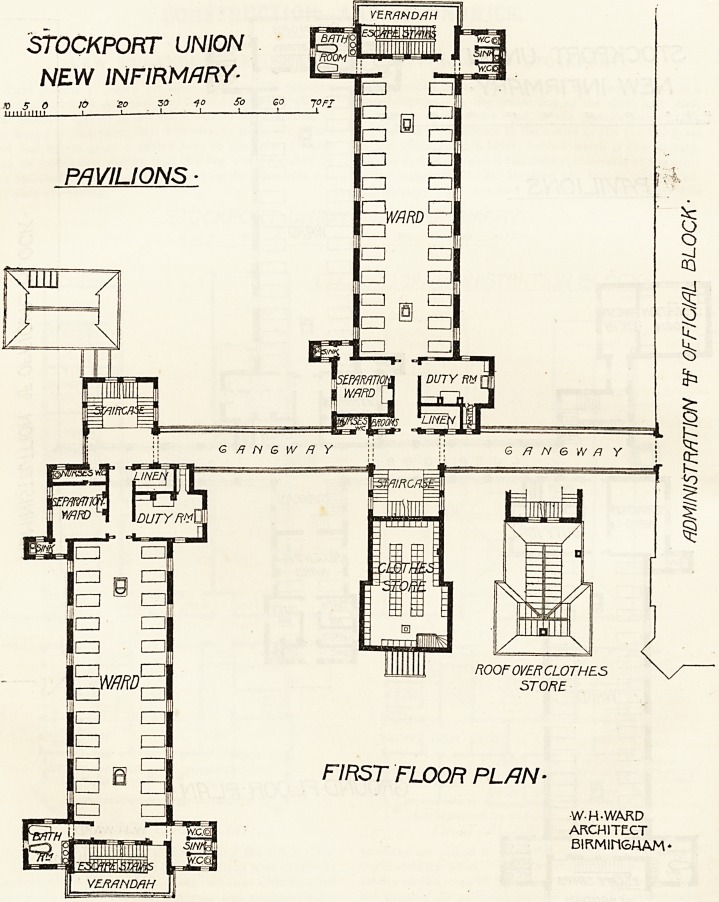# New Workhouse Infirmary at Stepping Hill, Stockport

**Published:** 1906-09-15

**Authors:** 


					428 THE HOSPITAL. Sept. 15, 1906.
HOSPITAL ADMINISTRATION.
CONSTRUCTION AND ECONOMICS.
NEW WORKHOUSE INFIRMARY AT STEPPING HILL, STOCKPORT.
This infirmary, which contains beds for 340 patients,
??accommodation for 36 nurses, and for 24 other indoor officials
was formally opened in December last by the Chairman of
the Board of Guardians, Mr. Andrews, to whom Mr. Ward
had just before given a golden key, on one side of which
was an inscription stating that the key was presented by
the architect and the contractor, and on the other side was
the date of opening.
Sixty-five years ago the workhouse and the old hospital
at Shaw Hill were built, and these buildings were originally
constructed for 554 inmates. Since that time additions had
been made for 330 inmates; but even this accommodation
was insufficient for the growing numbers; and it was stated
by Mr. Andrew that the workhouse and its adjuncts con-
tained nearly one thousand inmates; so that the new in-
firmary was not opened a day too soon. The infirmary con-
sists of nine distinct blocks and the operating theatre which
is attached to cne of the other blocks; and the immediate
site on which these units are placed has been laid out in a
parallelogram. In the centre line of the short axis, and,
of course, close to the road, is the porter's lodge; then
further back and almost in the centre of the parallelogram
is the administrative block, behind -which is the laundry.
At one end of the site is the nurses' home and at the other
is the maternity block. Between the former and the ad-
ministrative block are two pavilions, and the other two
pavilions are placed between the administrative bicck and
the maternity block. This arrangement, looking at it
merely on the score of convenience, seems decidedly good;
but whether the pavilions have, or could all possibly have,
the best aspects it is not easy to say, as the compass points
are not marked on the plans from which this notice is
written, and if the best aspects have not been obtained,
then the arrangement is bad, no matter how well it may
look on paper.
The administrative block is entered in the centre of the
STOCKPORT UNION . NEW INFIRMARY'
OFFICIAL If ADMINISTRATION BLOCK'
, . u .J -- v -- ^ & P y M ^ W P ill iL ? ? @ * b ^ ^ b ? ?
:? ?;::---- IP" W' Ij" 1 ?!-- ??- .. m?rpggaTmagagr? ...
P r?^ 6EHVIN6l\ r /> f5cRVIN6 ? ?| " T| ^ /x ?\ mi
U S~rrr-U. /\ /\ J In/spPNCggy}- * MATRONS ?-^-4 /\ f\ 'jrmnr XMFr-nmrM stewards
|4/7T/rQv|fnO OCT ti I KITCHEN I f_J ?jT[ KITCh'ES^???
|P?1 _ v_____v _ _
#T* f.
tt5?V///YS;: /.easy j; : -LOBBY H
m.?) _ jd   jj_ 1 .
^ T2pn/r?7/!r 1 I CHIEF TjlEDiCflLWr ?STEWARDS
RSUTIN6rda-MEDICf?L. .STE'.VAr.D'S*^ ROOM U 2MflTRON$* *M/]TROHS MEDICAL MEDICflk l/OFFICEftS1^?TT/ri6Ft*
ROOM LtMfFlCERST'' '1 room LJ *N JSITT<eR*^ ^BEDR" TF/CI^ 0FFICERS1^SITT1N6%$H ZVFUxm
s
ROOM
** ^ ALL BEDROOMS ON LV FLOOR ?j
GROUND FLOOR PL/IN- FIRST FLOOR PLAN-
Sept. 15, 1906. THE HOSPITAL. 420
elevation which faces the lodge, and on the left hand of the
vestibule are the medical officer's room, sitting-room, and
the serving-room. On the right hand are the steward's
office and committee-room. There is a fine, roof-lighted
waiting hall, on each side of which is a staircase; in front
is a lobby and behind it runs the main corridor of com-
munication. The matron's store-room occupies the space
alongside one of these staircases, and the dispensary the
space alongside the other one. Both of these have separate
passages opening into the main corridor. Beyond this
corridor are the kitchen, the nurses' mess-room, and various-
kitchen offices. At the end of these offices, but having no
communication with them is the disinfecting-room, which,
is fitted up with a Washington Lyons' disinfector. Running
STOCKPORT UNION
NEW INFIRMARY-
GROUND FLOOR PLAN ?
W-H WARD
ARCHITECT ?
BIRMIM&HAM ?
430 THE HOSPITAL. Sept. 15, 1906.
parallel with the kitchen is the general store-room, and
beyond this is the boiler-house.
The whole of this kitchen sub-division of the administra-
tive blook consists of ground floor only; but in front of the
main corridor the block is carried up to three stories. The
first floor contains rooms fo'r the medical officers and the
matron, and on the second floor are the steward's rooms,
servants' rooms, and spare accommodation.
The pavilion nearest the centre has attached to it, or
rather attached to the opposite side of the corridor, a re-
ceiving ward. This ward has its own approach from the
drive. It contains a ward for four beds, a nurses' duty
room, linen-room, bath-room, and closet. There is also a
staircase which leads to the first floor, and this is used as a
clothing store. The block therefore ensures that no patient
shall enter the pavilion proper until the nature of his
disease is definitely known, and his clothing will not bo
taken in at all. {To be continued.)
STOCKPORT UNION
NEW INFIRMARY-
PAVILIONS
FIRST FLOOR PLAN-
W-H-WARD
ARCHITECT
BIRMIM6MAM'

				

## Figures and Tables

**Figure f1:**
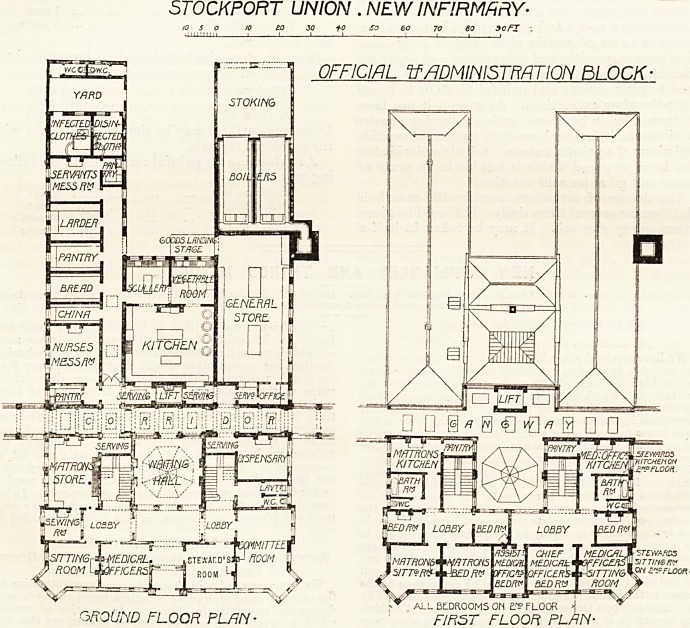


**Figure f2:**
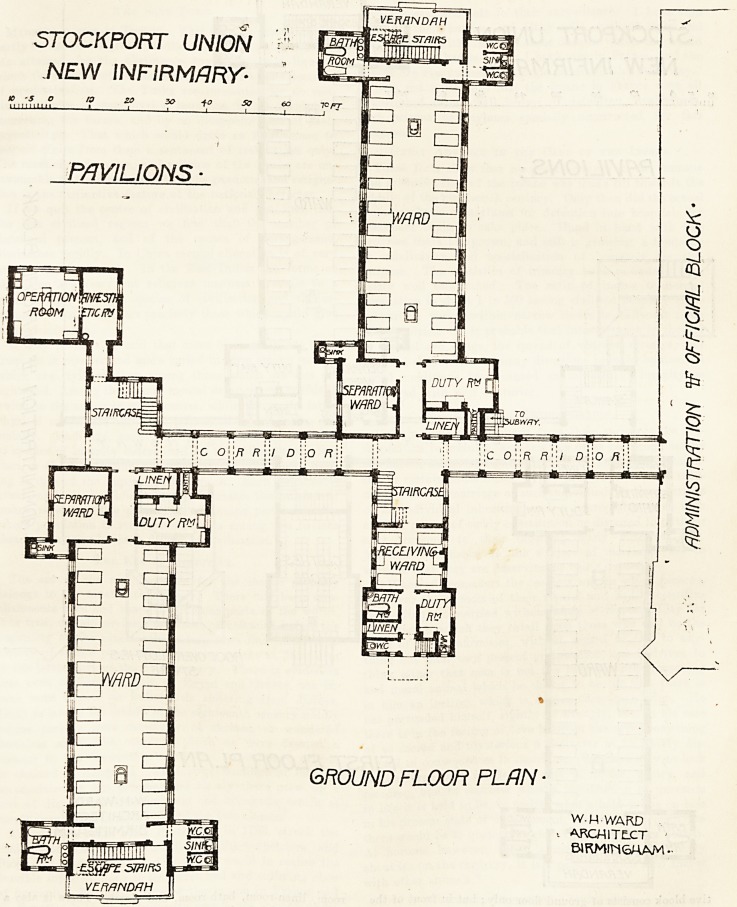


**Figure f3:**